# Impact of Floseal and NasoPore in Managing Epistaxis in the Emergency Department: A Quality Improvement Analysis

**DOI:** 10.7759/cureus.102043

**Published:** 2026-01-21

**Authors:** Usman Mansoor, Awais Cheema, Sarah Cowan-Rawcliffe, Maaza Mansoor, Lok Thapa

**Affiliations:** 1 Emergency Medicine, Al Jalila Children's Speciality Hospital, Dubai, ARE; 2 Emergency Medicine, University Hospitals Sussex NHS Foundation Trust, Sussex, GBR; 3 Emergency Medicine, Frimley Park Hospital, Camberley, GBR; 4 Medicine, Western Health, Melbourne, AUS; 5 Emergency Medicine, St. Peter’s Hospital, Chertsey, GBR

**Keywords:** bioresorbable hemostatic, ed length of stay, epistaxis management, nose bleeding management, patient satisfaction, quality improvement project

## Abstract

Background: Epistaxis is a common ear, nose, throat (ENT) presentation in the emergency department (ED), with the majority of the population having experienced an episode during their lifetime and required hospitalization. Like many district general hospitals (DGHs) in the country, St Peter’s Hospital does not offer adult inpatient or out-of-hours emergency cover for ENT, necessitating patients who require intranasal packing with a Rapid Rhino® (Smith & Nephew, Hull, UK) to be transferred to another hospital for assessment and admission, with most transfers being via ambulance. Apart from being resource-heavy, both the procedure itself and the prolonged stay in ED awaiting transport are a distressing experience for the patient. Therefore, the present quality improvement analysis was performed for the assessment of the effectiveness of Floseal® (Baxter Healthcare, Deerfield, IL, USA) and NasoPore® (Stryker, Kalamazoo, MI, USA), a bioresorbable material for epistaxis.

Methods: We introduced the revised Royal College of Surgeons (RCS) ENT UK guidelines for epistaxis management in our department. Two Plan-Do-Study-Act (PDSA) cycles were conducted in parallel. To assess the patient experience, a feedback form was designed with particular focus on pain score using the Visual Analog Scale (VAS). IBM SPSS Statistics for Windows, Version 16 (Released 2007; IBM Corp., Armonk, New York, United States) was used for descriptive analysis, and cost-effectiveness analysis was also performed to compare the outcomes with rapid rhino nasal packing. A telephonic follow-up on days 2 and 5 was organized to document any adverse features.

Results: The use of bioresorbable nasal packing among 60 patients demonstrated a clear benefit over the conventional practice of a non-absorbable packing, like Rapid Rhino. It reduced the length of stay (LOS) in ED and allowed the patients to go home with self-care instructions. In addition to much better patient comfort and tolerability (drop in pain score from 7 to 2 on VAS), this intervention also had a significant cost-saving impact with more than 65% cost reduction.

Conclusion: Floseal bioresorbable material can be used as a replacement for traditional nasal packing, as it was found to be more effective and cost-effective.

## Introduction

Epistaxis or nosebleed is the most common problem caused by the rupture of a blood vessel within the nasal mucosa or nasal cavity, nostril, or nasopharynx [1]. According to the current International Survey on epistaxis management, 21.7% prevalence was reported, with 11.8% hospitalization rate among children and the elderly population [2]. Likewise, the lifetime risk of developing epistaxis is estimated at 60%, but, as most cases are self-limiting, only 6% of patients with epistaxis seek medical attention [3,4]. Epistaxis occurring in adults aged >50 can be severe and originate posteriorly, whereas epistaxis occurring in children aged >10 years usually is of a mild nature and originates from the anterior nose (Kiesselbach's plexus), and an etiologic vessel can usually be found on careful nasal examination [5,6]. Rupture can be spontaneous, initiated by trauma to a systemic condition, use of certain medications, and/or secondary to other comorbidities or malignancies [7]. An increase in the patient’s blood pressure can increase the length of the episode. Anticoagulant medications, as well as clotting disorders, can also increase the bleeding time [1]. In addition, hyperlipidemia, hypertension, and alcohol consumption also cause epistaxis [8]. Meanwhile, in 80-90% of cases, epistaxis is anterior and can be managed with the application of proper compression techniques or topical applications [9]. In rare cases, it is associated with a significant morbidity or even death in a very rare situation [4]. Therefore, focus should also be centered on the management of epistaxis to avoid unnecessary events.

Traditionally, direct nasal pressure, suction, use of vasoconstrictors, cauterization with silver nitrate, packing with non-absorbable nasal tampons (Rapid Rhino®/Merocel®) removed at a later date (usually 48-72 hours later) under specialist care has been the mainstay of management in emergency settings [10,11]. Meanwhile, in the pre COVID-19 era, the management of epistaxis in the emergency department (ED) has been very similar across various trusts in the UK, aiming to control the bleeding with initially non-invasive methods such as manual nasal pressure, temporary intranasal packing with adrenaline/tranexamic acid soaked gauze and use of silver nitrate to cauterize the affected bleeding point [12]. If the bleeding continues, then the use of non-absorbable nasal packing such as Rapid Rhino® (Smith & Nephew, Hull, UK) (inflatable balloon tampon) or Merocel (compressed sponge tampon) has been advocated [1]. Post procedure disposition depends upon the availability of ear, nose, and throat (ENT) services, and the patient’s journey has mixed outcomes with eventual removal of the tampon after 48-72 hours under specialist supervision. However, such approaches, while found effective in controlling epistaxis, are frequently associated with risk of mucosal trauma, re-bleeding, infection, and patient discomfort. Therefore, safer and effective alternative approaches should be explored.

Recently, the development and clinical adoption of bioresorbable hemostatic materials, like Floseal® (Baxter Healthcare, Deerfield, IL, USA) and NasoPore® (Stryker, Kalamazoo, MI, USA), have revolutionized the management of epistaxis. Floseal is a gelatin-thrombin-based matrix sealant, which is designed to provide reliable and rapid hemostasis. It promotes the coagulation cascade through the thrombin component and also provides mechanical tamponade, thereby forming a stable clot at the bleeding site [13]. NasoPore is another synthetic, bioresorbable polyurethane-based material, which acts as a hemostatic scaffold and packing within the nasal cavity [14]. Meanwhile, post-COVID-19, the Royal College of Surgeons (RCS) ENT UK published its revised guideline for management of epistaxis [1], which calls for minimal steps towards the control of epistaxis in the ED, as a measure to prevent the spread of COVID-19. It incorporates the use of Floseal, if bleeding persists despite manual nasal pressure, while eliminating the use of vasoconstrictors, suctioning, and cautery as initial steps in the management of epistaxis in the ED.

Moreover, St Peter’s Hospital does not offer adult inpatient ENT services, nor out-of-hours emergency cover for ENT. The majority of our patients with epistaxis who present to our ED and end up requiring intranasal packing with a Rapid Rhino to control their bleeding are transferred over to Royal Surrey County Hospital (RSCH), Guildford, for assessment and admission under the care of the ENT department. Most of these transfers are ambulance transfers. Apart from being resource-heavy (approximately the cost of an ED stay plus ambulance transfer is up to £2000 per patient) [15], both the procedure itself and the prolonged stay in ED awaiting transport are a distressing experience for the patient [10]. Therefore, the present study aimed to evaluate the Floseal and NasoPore packing for the control of epistaxis.

Primary objectives

The primary objectives were to update the current ED epistaxis management guidelines in accordance with RCS ENT UK recommendations, and to assess patient comfort (pain) with the use of Floseal and NasoPore, soft gel-like bioresorbable materials, compared with conventional stiff nasal packing such as Rapid Rhino, as well as to compare the length of stay (LOS) in the ED.

Secondary objectives

The secondary objectives were to develop a patient information leaflet with clear home-care instructions, including do’s and don’ts following Floseal and NasoPore insertion; to create a standardized discharge information leaflet aimed at reducing referrals, transfers, and admissions to the RSCH Guildford ENT department; and to assess cost savings of approximately £2000 per patient by avoiding prolonged ED stays, ambulance transfers, and inpatient admissions.

## Materials and methods

Study design

This study was performed as a quality improvement initiative within the ED of St Peter’s Hospital, UK. It was based on the Model for Improvement and the Plan-Do-Study-Act (PDSA) framework [16], and we introduced the updated RCS ENT UK guidelines for epistaxis management in our ED [17]. With a focus on step-wise management of epistaxis in the ED and the use of Floseal and NasoPore instead of Rapid Rhino, the aim was not only to improve patient compliance with the procedure but also to assess their suitability for discharge home with advice rather than transfer to an ENT department at another hospital.

Eligibility criteria

For the selection of the participants, certain inclusion criteria were set, including only adult patients >18 years presenting with active epistaxis requiring intervention in the ED. Both anterior and posterior epistaxis were included. Likewise, certain exclusion criteria were also set for the selection of participants, including pediatric patients <18 years, patients requiring surgery under anesthesia, patients with major facial trauma or head injury, which required surgical procedures, individuals with known allergies to gelatin or thrombin components, and patients with active nasal tumors or infections.

Sample size

To measure the improvement in the patient’s journey, we did an internal analysis of our current practice. First, we performed a pilot study and included 20 patients with epistaxis presenting to the ED over a period of six months who required nasal packing with a Rapid Rhino. After a pilot study, we included 60 patients for a period of 10 months.

Intervention

Floseal and NasoPore were introduced for the management of epistaxis in the ED with the intention of achieving improvement and a cost-effective approach.

Production preparation and application

Floseal was prepared by reconstituting the gelatin matrix with human thrombin according to the instructions provided by the manufacturer. Briefly, the lyophilized human thrombin was dissolved in the provided diluent to obtain the recommended concentration, after which the thrombin solution was transferred into the syringe containing the gelatin matrix using the supplied connector. The components were gently mixed by repeatedly transferring the contents between syringes until a homogeneous, flowable hemostatic matrix was achieved, ensuring the absence of air bubbles.

**Figure 1:**
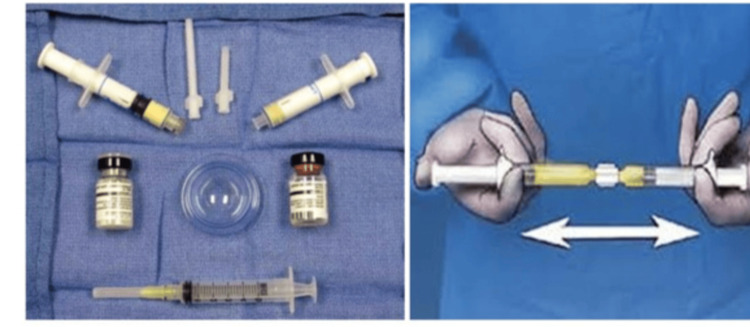
Preparation of Floseal and its application for the control of epistaxis. Credit: The image in the right panel was developed by the authors using Canva (Canva Pty Ltd., Sydney, Australia; canva.com).

When the source of bleeding was not readily identified, flexible fibreoptic nasendoscopy was performed to determine the level of bleeding. After localization, one unit (3-5 mL per nostril) of Floseal was applied using the appropriate applicator directly to the bleeding point until hemostasis was achieved (Figure 1), and the remaining gel was used to fill the ipsilateral nasal cavity.

**Figure 2:**
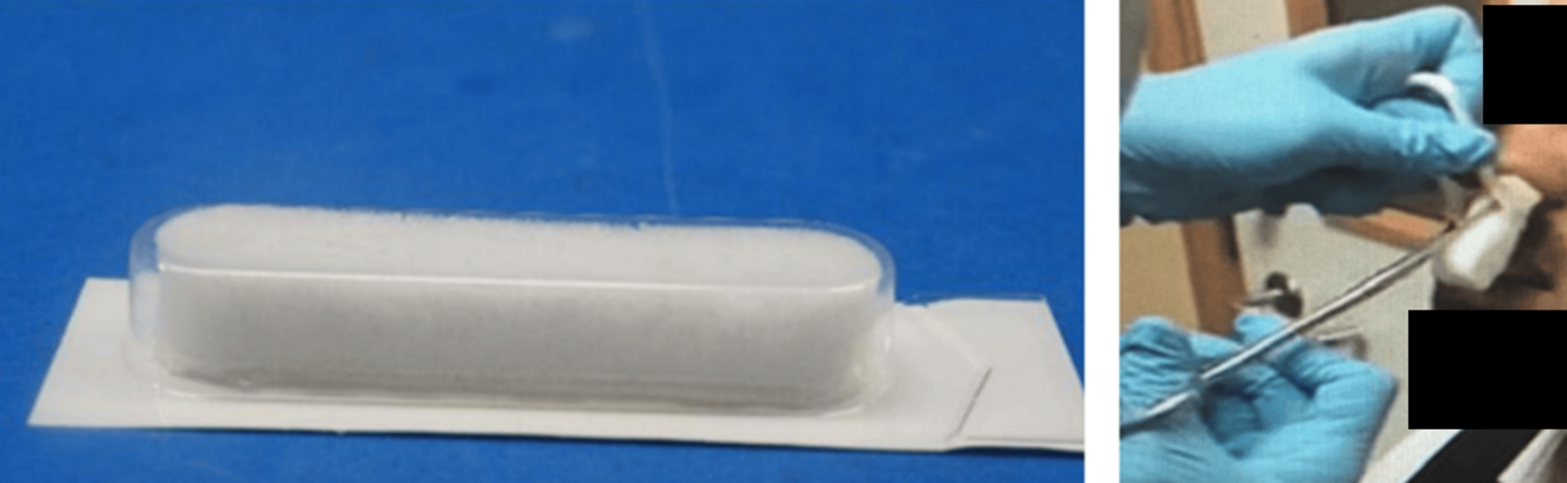
Insertion of NasoPore over Floseal using Tilley forceps.

NasoPore, a standardized piece of 8 cm material, was used as an adjunct packing material, and it was inserted into the nasal cavity after ensuring adequate hemostasis with Floseal (Figure 2). Hemostasis is generally assessed within 5-10 minutes after application, with repeat inspection performed at 20-30 minutes to confirm sustained bleeding control. Failure criteria prompting escalation to a fallback intervention, such as Rapid Rhino, typically include persistent active bleeding after a second Floseal application, rebleeding during the observation period, inability to achieve visualization due to ongoing hemorrhage, or hemodynamic instability requiring rapid definitive nasal packing.

Implementation strategy

PDSA Cycle 1

Initial audit findings alongside updated COVID-19 epistaxis management guidelines by RCS ENT UK were discussed amongst the core team members, and the need for a bioresorbable nasal packing as an alternative to Rapid Rhino was shared. Due to the cost of the Floseal (£200 per piece), a short business case was written up for us to pilot its use and demonstrate effectiveness. After the approval from the ED service manager, the order was placed with the help of the ED pharmacist, and a total of 10 packs of Floseal were received.

PDSA Cycle 2

A second bioresorbable agent, NasoPore, was introduced to be used in conjunction with Floseal in the management of uncontrolled epistaxis. NasoPore, being bioresorbable, shares similar characteristics to the Floseal; however, its appropriate insertion into the nasal cavity can be the limiting factor. Hence, it is considered a second-line treatment option when compared to Floseal. In uncontrolled epistaxis, including posterior bleeds, we decided to insert Floseal in the posterior space, followed by insertion of NasoPore to fill the nasal cavity. NasoPore will not only serve as a seal to allow Floseal to set in the space, but will also play a role to augment hemostasis by creating a tamponade effect.

Data collection

The following data were extracted from the study participants: patient experience with the use of Floseal and NasoPore as alternatives to Rapid Rhino in the management of epistaxis, including pain scores measured using the Visual Analog Scale (VAS) [18], length of hospital stay, and the cost of each procedure performed. In addition, patient information leaflets were prepared to illustrate general management guidance and post-procedure care following Floseal insertion. A patient feedback form was designed to record the experience during the procedure, and telephone follow-up was arranged on days 2 and 5 to document any adverse events.

Data analysis

Data was analyzed using IBM SPSS Statistics for Windows, Version 16 (Released 2007; IBM Corp., Armonk, New York, United States), and descriptive analysis was performed for the presentation of data in frequencies, while graphs were created using Microsoft Excel 2010 (Microsoft Corp., Redmond, WA, USA). Pain scores were calculated using VAS scores [18], and cost-effectiveness analysis was conducted by systematically collecting and comparing direct and indirect cost data associated with the use of Floseal. Direct medical costs included the acquisition cost of Floseal, operative time, additional hemostatic agents, blood transfusions, management of bleeding-associated complications, and LOS, while indirect costs, where reported, encompassed resource utilization related to postoperative care.

## Results

Pilot study

Among 20 patients in the pilot study, the feedback was overwhelmingly positive, and the simplicity of the procedure stood out. The initial pilot study demonstrated the need for wider training in the future for the initial preparation of the Floseal from the box, as it included a few steps to achieve the final product in the syringe. Mainly, two reasons were identified that resulted in failure to control epistaxis with the use of Floseal in the initial pilot study. Some spillages of Floseal material were noted out of the affected nostril immediately after its insertion if appropriate measures were not taken. Advised to have a minimal head tilt back whilst sitting upright on the trolley and also use of a "mustache dressing" with the help of a rolled-up gauze (nasal bolster swab dressing) to minimize the initial leak of the Floseal, as it can take up to a few minutes for the Floseal gel to set inside the nasal cavity. Failure to control posterior nasal bleeds, if not identified in the beginning, will require adequate filling of the posterior space.

PDSA 1

**Figure 3:**
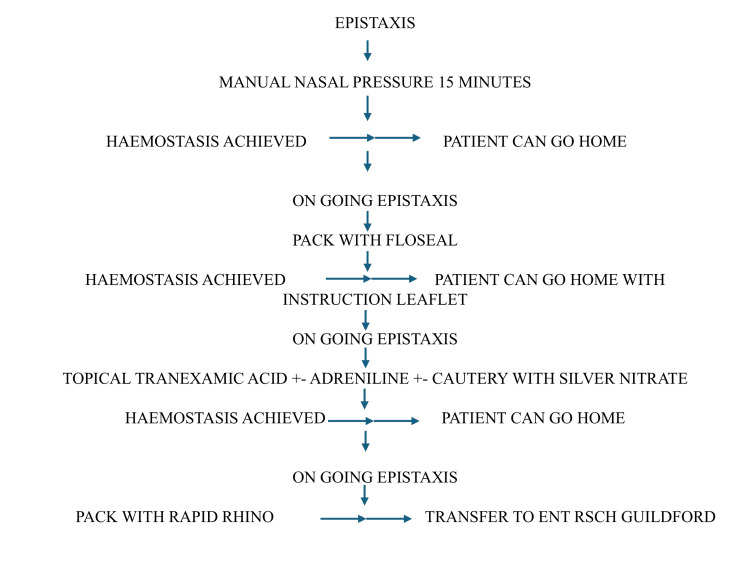
PDSA cycle 1 guidelines for the management of epistaxis using Floseal. PDSA: Plan-Do-Study-Act; RSCH: Royal Surrey County Hospital

The feedback received was overwhelmingly positive, and the simplicity of the procedure stood out. Initial analysis of the data demonstrated that the information leaflet for PDSA cycle 1 was developed and is explained in Figure 3. This figure describes a stepwise algorithm for managing epistaxis in the ED, emphasizing a progressive and evidence-based approach to achieving hemostasis.

PDSA 2

Based on the findings and experience of PSDA 1, management of posterior bleeds remained a challenge, as few cases were encountered where there was evidence of both anterior and posterior nasal bleeds, and Floseal alone was unable to achieve hemostasis. Aftercare with NasoPore slightly differs when compared to Floseal. Nasal irrigation twice a day with saline for the first two to four weeks is advised, which will enhance fragmentation, followed by a trip to the ENT clinic to ensure it has completely dissolved. In some instances, it might require gentle suction to remove any remaining particles of the NasoPore after four to six weeks, which is done at a local ENT clinic follow-up. Given the soft texture and its bioresorbable qualities, it is still acceptable for the patient to go home with self-care instructions.

The following salient points were noted after the change: (1) modification of the epistaxis guidelines with the addition of NasoPore for uncontrolled bleeding to be used in conjunction with Floseal; and (2) documentation of pain scores using the VAS for Floseal and NasoPore, with comparison to Rapid Rhino.

**Figure 4:**
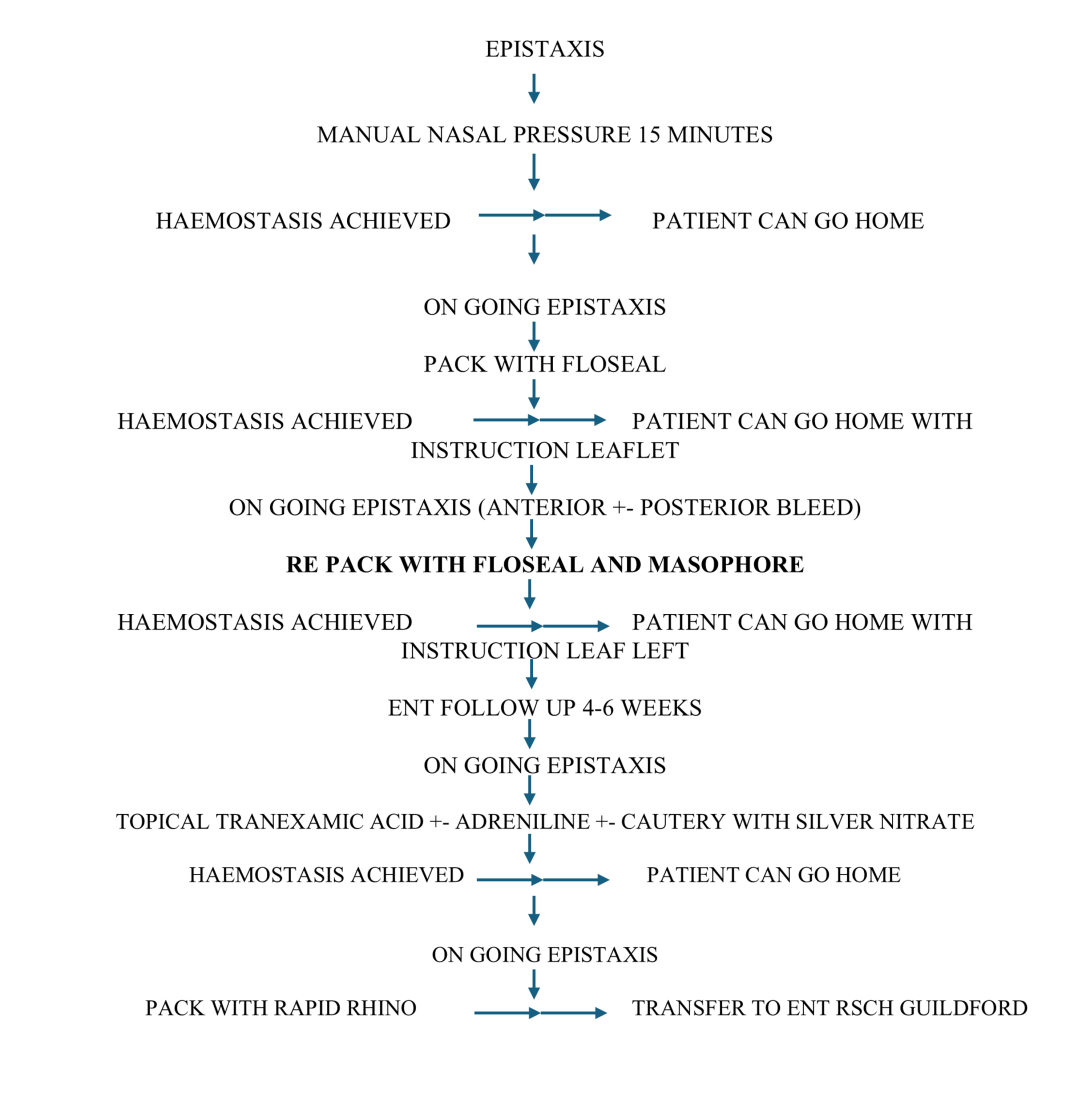
PDSA guidelines for the management of epistaxis using both Floseal and NasoPore. PDSA: Plan-Do-Study-Act; RSCH: Royal Surrey County Hospital

The information leaflet for PDSA cycle 2 was developed and is presented in Figure 4.

Combine effects

In the following month, Floseal and NasoPore were trialed on two cases where bleeding failed to cease with Floseal alone. Results were promising, and we achieved hemostasis; however, cost-effectiveness was reduced as a second pack of Floseal had to be used along with NasoPore with a subsequent follow-up at the ENT clinic. Both cases were successfully discharged home after a brief period of observation, highlighting the benefit of rapid rhino.

Pain scores

**Figure 5:**
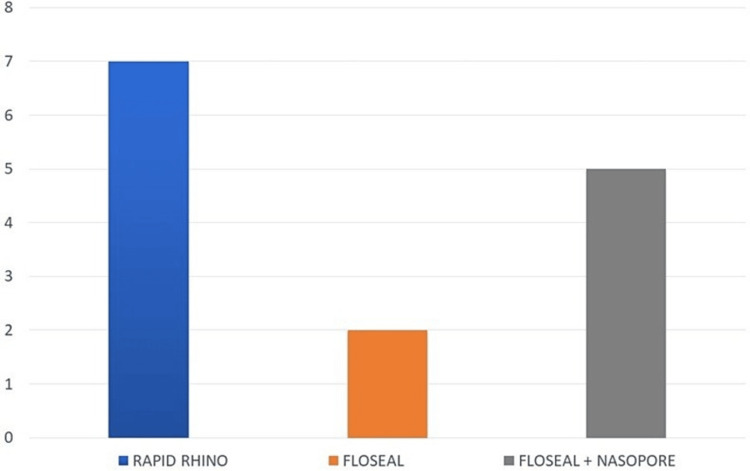
Pain scores among patients treated with Floseal alone, Floseal + NasoPore, and Rapid Rhino, assessed using the Visual Analog Scale (VAS).

The patients undergoing packing with Rapid Rhino reported a pain score of 7 on the VAS, compared to a pain score of just 2 with the use of Floseal alone. Thus, the use of Floseal was associated with a demonstrable reduction in pain. In the minority of patients who required both Floseal and NasoPore for the management of epistaxis, the pain score was reported as 5. This was still a significant reduction in pain compared to the use of Rapid Rhino (Figure 5).

Length of hospital stay

**Figure 6:**
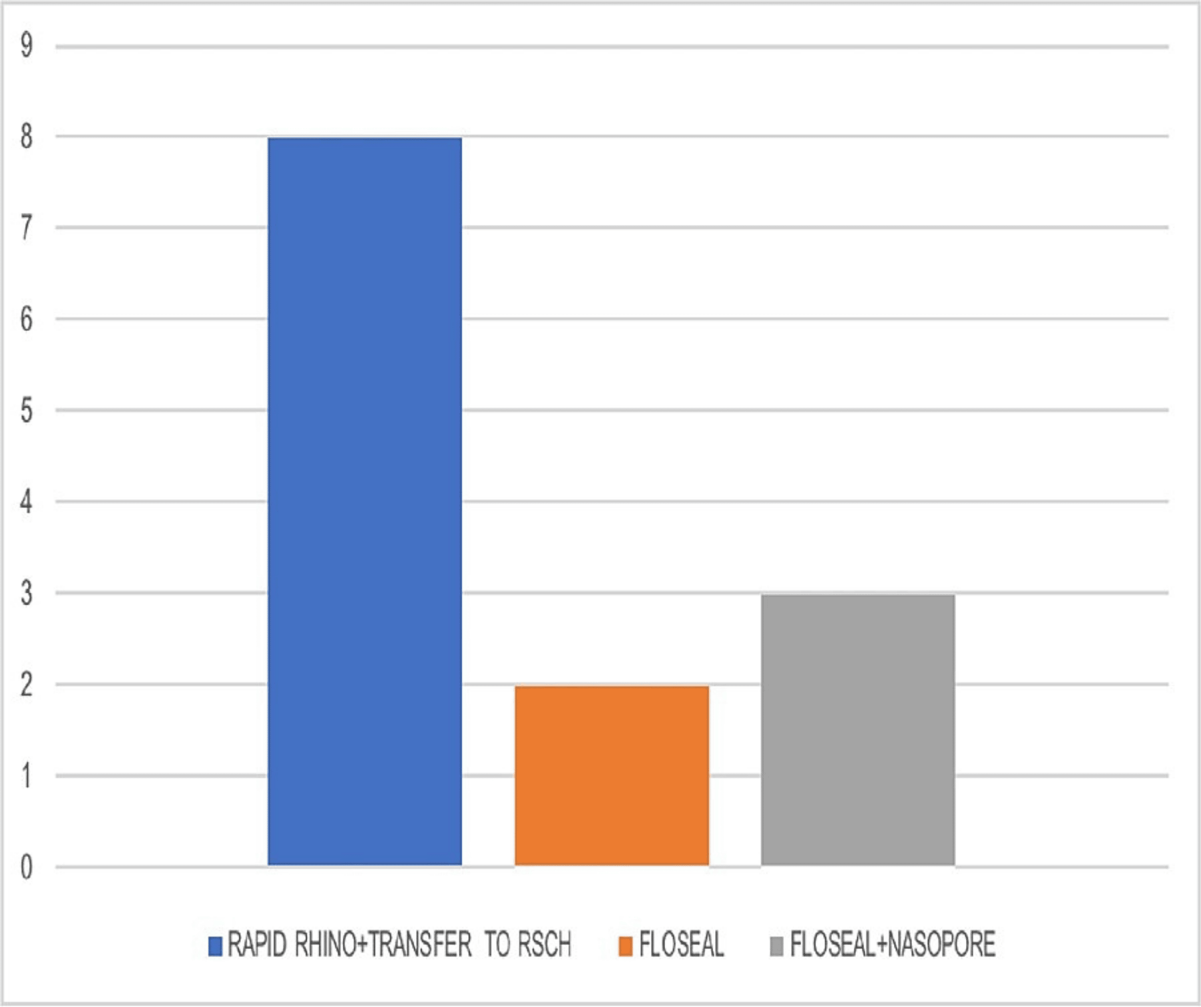
Emergency department length of stay in patients treated with different interventions for the control of epistaxis. RSCH: Royal Surrey County Hospital

The average ED LOS for patients who required insertion of Rapid Rhino and onward transport to another hospital for further inpatient management was eight hours in our department. Compared to this, the LOS for patients undergoing Floseal insertion was remarkably reduced to a maximum of two hours. This reduction not only had a hugely positive impact on patient satisfaction but also freed up the space and personnel to see more patients. The LOS for the limited number of patients requiring both Floseal and NasoPore insertion was also significantly reduced at three hours (Figure 6).

Cost-effectiveness

**Figure 7:**
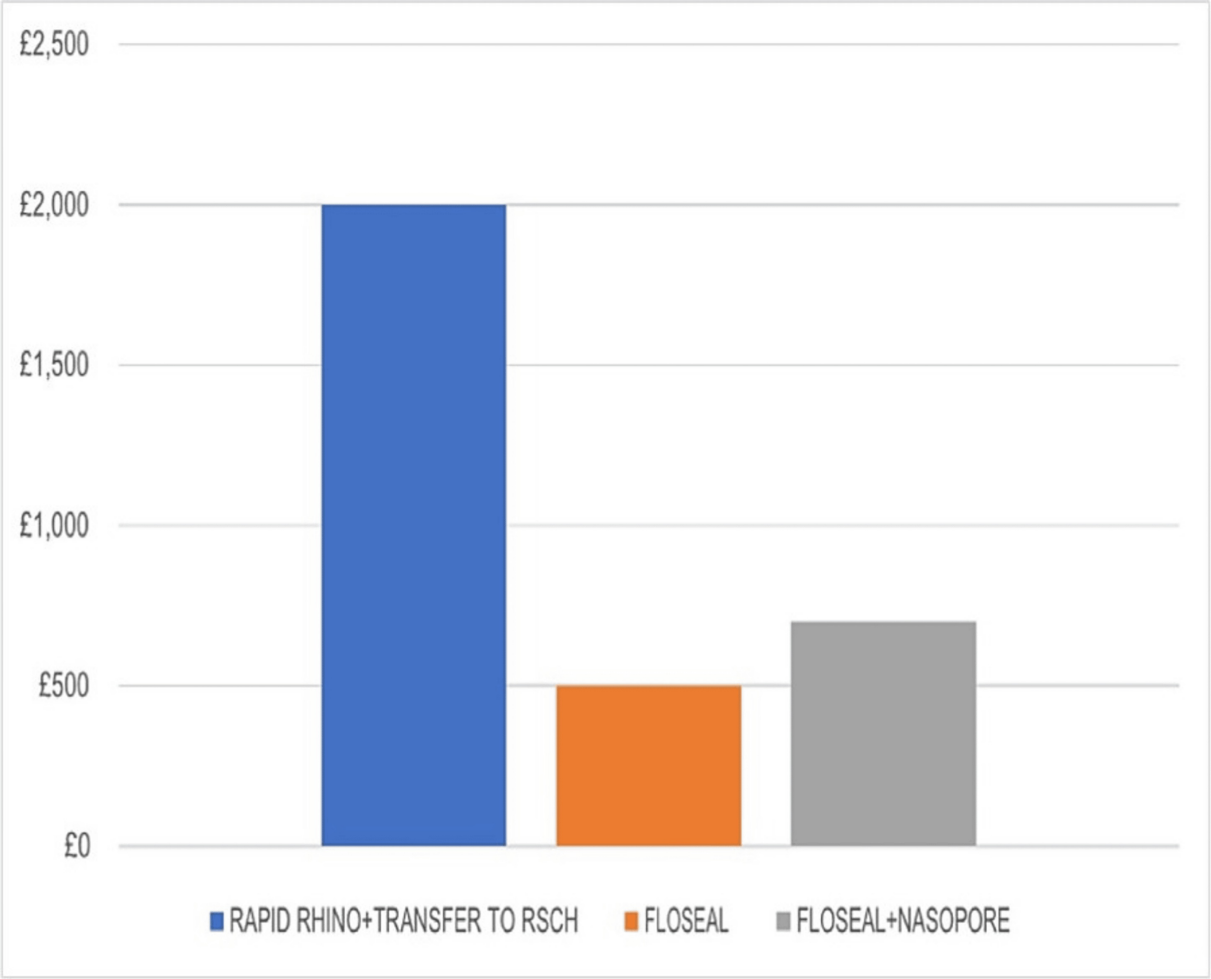
Cost-effectiveness analysis of each intervention. RSCH: Royal Surrey County Hospital

The approximate cost of each patient who required insertion of Rapid Rhino and then needed transfer to another hospital was 2000£. With the use of Floseal, the total cost throughout the patient’s treatment was significantly reduced to 500£ per patient. This represents a more than 65% cost reduction for the department for such patients (Figure 7).

## Discussion

We conducted this quality improvement project, examining the use of Floseal and NasoPore in patients presenting to the ED with epistaxis. Since the start of this project, we have used Floseal alone or in combination with NasoPore in 60 patients over 10 months with excellent results. Both Floseal alone and in combination with NasoPore had demonstrated a significant improvement in pain score, cost, and LOS, compared to the Rapid Rhino. For pain assessment, VAS was used, which is a useful measure previously used to determine pain in patients with epistaxis undergoing nasal packing with Rapid Rhino and other nasal packings [19].

Our study clearly demonstrated a drop in pain score from 7 to 2 on VAS for Floseal alone, which advocates a much better-tolerated intervention. In addition, no adverse effects had been reported. Overall, the use of bioresorbable materials has shown a great deal of promise for the management of epistaxis in our clinical settings. Similar findings were observed in a randomized trial, which compared NasoPore with a non-resorbable traditional impregnated gauze packing group. NasoPore group patients had higher patient comfort, and mucosal healing was also high [20]. Similarly, another study demonstrated a high success rate (90%) for Floseal alone, with a high level of patient satisfaction (8.4/10 points), and LOS was 2.75 hours [21]. Another prospective study also reported a high success rate (80%) when patients were treated with Floseal [22]. Likewise, another randomized trial also reported a significant (p=0.002) decrease in pain (2.42 vs. 7.77) compared to the control group and also provided an average saving of 1567.61 $/patient; however, uncertainty analysis indicated that Floseal had a 90% chance of not being cost-effective, but dominant and preferred treatment option [23]. Another economic evaluation for Floseal also suggested that Floseal was associated with a higher cost (2067 $), but with a greater quality-adjusted life year (QALY) of 0.27 compared to nasal packing [24]. Meanwhile, the economic burden of any intervention in ED is a reality, and all efforts should be directed towards the most cost-effective product. More than 65% of the cost savings observed in our study is a significant amount when one considers the sheer number of patients with epistaxis that present every year to UK EDs. The observed 65% cost saving likely reflects the broader economic efficiency of using Floseal and NasoPore compared to Rapid Rhino nasal packing, when considering the full spectrum of healthcare resource utilization. While the initial cost of the product may be higher, it substantially reduces the indirect cost by minimizing the requirement of hospital admission, shortening the time in ED, lowering or eliminating complications, and successfully avoiding the need for painful pack removal and follow-up visits. Moreover, it has been reported that older patients with multiple comorbidities need more inpatient management for epistaxis and overall need for a strategy to reduce this period [25]. Use of Floseal and NasoPore has led to reduced length of inpatient stay, resulting in much improved patient satisfaction. The patients who underwent Floseal insertion also did not need a routine ENT follow-up appointment, thus relieving pressure on stretched specialty services. Similar outcomes were reported by Mathiasen and Cruz [26], and they concluded that Floseal was found effective and more satisfying when compared to the traditional nasal packing, and also observed no adverse events or re-bleeding during the follow-up.

Clinically, our findings suggest the incorporation of Floseal and NasoPore into the standard epistaxis management protocols in the ED, as it can enhance the patient’s satisfaction by reducing the pain and streamlining clinical workflow. These advantages ultimately resulted in shorter LOS in ED, minimized follow-up visitations, fewer hospital admissions, and, most importantly, reduced the workload of the staff.

The main strength of our study was the use of a real-world quality improvement design. This reflects practical implementation within the ED setting and also provides direct clinical significance in terms of effectiveness and cost-effectiveness. However, the study also had certain limitations, such as the inclusion of a small sample size and single-centered settings, which may limit the generalizability of the findings. Another important limitation is the lack of randomization, which may introduce bias. Despite these limitations, the findings of our study provide strong evidence supporting the feasibility and benefits of adopting Floseal and NasoPore bioresorbable materials in routine ED practice for the management of epistaxis.

## Conclusions

This quality improvement study has clearly demonstrated the benefits of a bioresorbable nasal packing over the conventional use of a non-absorbable packing, like Rapid Rhino. Not only was there a huge improvement in patients' tolerability with the use of Floseal alone, but we also achieved significant cost savings for the department. With the current ever-mounting pressures on ED, this mode of treatment suits both the patients and the departments by reducing the LOS in the ED while ensuring safe disposition. A future, prospective, randomized multicenter study with a large sample size is required for the validation of our findings.
